# Refractory myasthenia gravis treated with autologous hematopoietic stem cell transplantation

**DOI:** 10.1002/acn3.52246

**Published:** 2024-12-31

**Authors:** Benjamin Beland, Jan Storek, Liam Quartermain, Christopher Hahn, C. Elizabeth Pringle, Pierre R. Bourque, Michael Kennah, Natasha Kekre, Christopher Bredeson, David Allan, Kareem Jamani, Christopher White, Harold Atkins

**Affiliations:** ^1^ Division of Neurology, Department of Clinical Neurosciences, Cumming School of Medicine University of Calgary Calgary Alberta Canada; ^2^ Division of Hematology, Department of Medicine, Cumming School of Medicine University of Calgary Calgary Alberta Canada; ^3^ Transplant and Cell Therapy Program, Division of Hematology, Department of Medicine The Ottawa Hospital Ottawa Ontario Canada; ^4^ Division of Neurology, Department of Medicine, Faculty of Medicine University of Ottawa Ottawa Ontario Canada

## Abstract

**Objectives:**

Patients with refractory myasthenia gravis (MG) have few treatment options. Autologous hematopoietic stem cell transplantation (HSCT) has been used to treat immune diseases; however, its use in the treatment of MG is not broadly considered. Our objective is to report on the efficacy and safety of HSCT in refractory MG.

**Methods:**

Twenty‐one patients who underwent HSCT for MG were retrospectively reviewed. All patients had severe MG refractory to multiple therapies. Stem cells were mobilized with cyclophosphamide and granulocyte colony‐stimulating factor. The grafts were depleted of immune cells by selecting CD34+ cells. HSCT conditioning consisted of high‐dose cytoreductive therapy and anti‐thymocyte globulin. The primary efficacy outcome was achieving clinically stable remission or minimal manifestations without treatment and remaining as such until most recent follow‐up.

**Results:**

The median time from MG diagnosis to HSCT was 4.0 years. The primary outcome was reached in 16 of 18 evaluable patients (89%) at a median of 1.7 years and maintained with a median follow‐up of 6.7 years (range 1.0–21.9 years). Three patients were not evaluable for the primary outcome: one due to confounding illness and two died within 12 months of transplant. The transplant‐related mortality at 100 days was 9.5%. Two late deaths occurred, with uncertain relation to the HSCT.

**Interpretation:**

After HSCT for refractory MG, most patients achieved sustained disease remission. However, HSCT‐related mortality in medically complex MG patients may be high. Prospective studies investigating the efficacy and safety of HSCT in the treatment of refractory MG are warranted.

## Introduction

Myasthenia gravis (MG) is an autoimmune neurologic condition characterized by an antibody‐mediated disruption of neurotransmission and remodeling of the neuromuscular junction.^1^ Patients with MG experience fatigable weakness, typically affecting skeletal muscles including muscles controlling the eyes, speaking, swallowing, breathing, and appendicular muscles.^1^ Disease‐modifying therapy is aimed at suppressing the immune system. Apart from cholinesterase inhibitors, mainstay therapies include oral prednisone and steroid‐sparing agents such as azathioprine and mycophenolate mofetil.^1^ However, approximately 8–15% of patients continue to experience symptoms despite treatment with one or more of these agents.^2^, ^3^ For these individuals, additional therapies are used, including plasma exchange, intravenous immunoglobulin, cyclophosphamide, B cell‐depleting agents like rituximab, complement inhibitors like eculizumab or ravalizumab, neonatal Fc receptor (FcRn) inhibitors like efgartigimod, or thymectomy in selected patients.^1^, ^4^, ^5^ Patients are considered to have refractory MG (rMG) when MG symptoms persist even with the use of corticosteroids plus two additional immunomodulating/immunosuppressive agents given at appropriate doses for an adequate duration.^6^ Patients with rMG are three times more likely to experience myasthenic crisis, and more than two times as likely to die from their disease compared to patients without rMG.^7^ For patients whose disease is not controlled by these therapies or for those who experience intolerable side effects, few treatment options exist.

Autologous hematopoietic stem cell transplantation (HSCT) typically consists of conditioning with high‐dose lymphocytotoxic chemotherapy and lympho‐depleting antibody therapy, followed by an infusion of previously collected autologous hematopoietic stem cell grafts to minimize the duration of chemotherapy‐induced agranulocytosis and to effectively “reset” the immune system.^8^ HSCT has been used in the treatment of severe immune‐mediated diseases such as systemic sclerosis,^9^, ^10^ multiple sclerosis,^11^, ^12^ and Crohn's disease.^13^, ^14^ The mechanism of action may involve removing or inhibiting auto‐reactive T or B cells, including those that mediate the interference with or the destruction of the neuromuscular junction.^14^, ^15^ Previous case reports or small case series with relatively short follow‐up have described individuals undergoing HSCT for treatment of refractory generalized MG.^16^, ^17^, ^18^, ^19^, ^20^, ^21^, ^22^ Here we present a series of 21 consecutive patients from two tertiary care centers, with up to 22‐year post‐transplant follow‐up.

## Subjects, Materials, and Methods

### Patients

Cases of primary generalized MG were identified retrospectively from the databases of the Alberta Bone and Marrow Transplant Program (Calgary, Alberta, Canada) and The Ottawa Hospital Transplant and Cell Therapy Program (Ottawa, Ontario, Canada). All patients were identified by their treating neurologist as having rMG (failure to achieve adequate symptom control resulting in an impact on quality of life with the use of corticosteroids plus at least two additional immunomodulating/immunosuppressive agents given at appropriate doses for an adequate duration) and referred to hematology for an assessment of eligibility for transplant. All patients who underwent HSCT between January 2001 and December 2022 were included in the study. MG was diagnosed via clinical assessment, electrophysiology including repetitive nerve stimulation testing, and serum antibody testing for anti‐acetylcholine receptor antibodies (AChR) and anti‐muscle‐specific kinase (MuSK) antibodies (Table 1). Extended follow‐up is reported on 10 patients that were reported in two previous publications (Table S1).^16^, ^19^

**Table 1:** Clinical demographics and characteristics of myasthenia gravis.

Patient no.	Sex (M/F)	Age at diagnosis (years)	Age at HSCT (years)	Salient comorbidities
1	F	58	62	Ulcerative colitis
2	F	61	64	Hepatitis B, pernicious anemia, fibromyalgia
3	F	52	55	Celiac disease, pernicious anemia
4	F	35	38	None
5	F	54	56	Anxiety, osteoporosis with fractures, history of Covid/organizing pneumonia x2
6	F	38	43	Grave's disease, asthma
7	M	43	54	Psoriatic arthritis
8	F	35	49	None
9	F	37	42	Hashimoto thyroiditis, systemic lupus erythematosis
10	F	18	25	Congenital incontinenia pigmenti
11	F	35	42	Hashimoto thyroiditis, celiac disease
12	M	30	42	Sciatica, eczema
13	M	39	41	None
14	M	63	65	None
15	M	46	48	None
16	M	55	58	None
17	F	33	55	MCTD
18	F	56	58	None
19	F	41	43	Hashimoto thyroiditis, erythema nodosum
20	F	24	41	MCTD, anxiety
21	F	37	43	None
Median		39	48	

HSCT, autologous hematopoietic stem cell transplant; MCTD, mixed connective tissue disease.

The shading was to signify an empty cell.

### Hematopoietic stem cell transplant methods

Autologous hematopoietic stem cells were mobilized from bone marrow into the circulation using cyclophosphamide and filgrastim and harvested by mononuclear cell apheresis. The apheresis product (graft) of most patients was depleted of immune cells using immunomagnetic enrichment of CD34^+^ cells (CliniMACS, Miltenyi). The majority of patients (*N* = 17) received transplant conditioning with busulfan, cyclophosphamide, and rabbit anti‐thymocyte globulin, a regimen used also for multiple sclerosis^23^ and reported in our previous series on MG.^19^ Four patients received a different conditioning regimen (see Table 2). The choice of conditioning therapy is individualized based upon the patient characteristics in order to minimize risk and achieve optimal disease control. The choice is based on the transplant team experience, the reported outcomes in the medical literature, and physician opinion as well as patient factors including disease indication, prior exposures, comorbidities, and frailty. Differences in the opinion of the treating team resulted in heterogeneity of HSCT regimens used in the cohort. At least 2 × 10^6^/kg CD34 cells were infused on Day 0.

**Table 2:** Hematopoietic stem cell transplant details.

Patient number	Year of transplant	Time from Dx to HSCT (years)	Stem cell mobilization	CTX mobilization dose (g/M2)	Graft CD34 selected? (Y/N)	CD34 cell count (10^6^ cells)	CD3 cell count infused (10^6^ cell #/kg)	Conditioning regimen; (mg/kg, × days)	ICU peri‐transplant^a^	HSCT hospitalization (days)	Complications during HSCT hospitalization	Complications from discharge to 100 days post‐transplant	Late transplant complications (>100 days post‐transplant)
1	2017	3.8	CTX + GCSF	2.5	Y	4.20	ND	BU (2.4 × 4) CTX (50 × 4) ATG (4.5^c^)	N	21	FN	–	–
2	2018	3.5	CTX + GCSF	2.5	Y	6.30	ND	BU (2.4 × 4) CTX (50 × 4) ATG (4.5^c^)	N	37	FN	FN, sepsis	–
3	2018	3.1	CTX + GCSF	2.5	Y	5.40	ND	BU (2.4 × 4) CTX (50 × 4) ATG (4.5^c^)	N	23	FN	–	–
4	2020	2.9	CTX + GCSF	2.5	Y	7.80	ND	BU (2.4 × 4) CTX (50 × 4) ATG (4.5^c^)	N	22	FN	FN	Premature menopause, transient anemia
5	2022	2.1	CTX + GCSF	2.5	Y	4.00	ND	BU (2.1 × 4) CTX (50 × 4) ATG (4.5^c^)	Y	25	*E. coli* bacteremia, heart failure, vertebral compression fractures	Rehospitalized, including ICU: CMV viremia, BKV and enterococcal cystitis, staph epi sepsis, pneumonia not responding to ganciclovir + antibacterials, death from respiratory failure^b^	N/A
6	2001	4.8	CTX + GCSF	1.5	Y	8.95	Below level of detection	CTX (50 × 4) TBI (3 Gy × 1) ATG (1.25 × 4)	Y	43	N/A	ICU admission	Dyspnea
7	2007	11.3	CTX + GCSF	4.5	Y	4.20	Below level of detection	CTX (50 × 4) TBI (3 Gy × 1) ATG (1.25 × 4)	N	19	Myasthenic crisis without ICU admission	–	Died from adenocarcinoma colon
8	2010	14.7	CTX + GCSF	4.5	Y	2.74	0.01	BU (2.4 × 4) CTX (60 × 2) ATG (1.6 × 3)	N	37	–	–	–
9	2010	5.0	CTX + GCSF	2.5	Y	3.04	Below level of detection	BU (2.4 × 4) CTX (50 × 4) ATG (1.25 × 4)	N	34	–	–	Crohn's colitis, amegakaryocytic thrombocytopenic purpura, diarrhea, immune thrombocytopenic Purpura
10	2011	6.9	CTX + GCSF	1.5	Y	3.72	Below level of detection	BU (2.4 × 4) CTX (50 × 4) ATG (1.25 × 4)	N	29	FN, esophagitis	–	–
11	2011	7.3	CTX + GCSF	2.5	Y	5.89	Below level of detection	BU (2.4 × 4) CTX (50 × 4) ATG (1.25 × 4)	N	24	FN	CMV viremia, treated with gangciclovir, BKV cystitis treated with cidofovir	Saddle PE, sepsis
12	2015	12.1	CTX + GCSF	2.5	Y	4.34	Below level of detection	BU (2.4 × 4) CTX (50 × 4) ATG (1.25 × 4)	N	35	Epididymitis, dysuria, GERD	‐	Hypothyroid, cholecystectomy, umbilical hernia
13	2017	2.2	CTX + GCSF	2.5	Y	2.40	Below level of detection	BU (2.4 × 4) CTX (50 × 4) ATG (1.25 × 4)	N	32	FN	–	–
14	2019	2.0	CTX + GCSF	2.5	N	2.30	Below level of detection	BCNU (300 × 1) Etoposide (200 × 4) Cytarabine (1600 × 4) Melphalan (140 × 1) ATG (1.25 × 4)	Y	16	FN, refractory enterobacter septic shock requiring ICU admisision resulting in death	–	N/A
15	2019	2.2	CTX + GCSF	2.5	Y	3.34	Below level of detection	BU (2.4 × 4) CTX (50 × 4) ATG (1.25 × 4)	N	40	FN, CMV viremia	CMV viremia, treated with gangciclovir	Hypothyroid, cholecystectomy
16	2019	2.8	CTX + GCSF	2.5	Y	9.12	0.01	BU (2.4 × 4) CTX (50 × 4) ATG (1.25 × 4)	Y	43	FN, PE, C. Diff, gastric ulcer	–	Kidney stone
17	2021	21.8	CTX + GCSF	2.5	Y	5.21	0.01	BU (2.4 × 4) CTX (50 × 4) ATG (1.25 × 4)	Y	43	FN and sepsis requiring ICU admission	–	Immune thrombocytopenic purpura, T11 fracture
18	2022	1.9	CTX + GCSF	2.5	Y	7.94	0.01	BU (2.4 × 4) CTX (50 × 4) ATG (1.25 × 4)	N	39	FN, staphyoloccocus bacteremia	FN	–
19	2022	2.0	CTX + GCSF	2.5	Y	5.64	Below level of detection	BU (2.4 × 4) CTX (50 × 4) ATG (1.25 × 4)	N	33	CMV Viremia	CMV viremia, treated with valganciclovir	–
20	2022	19.2	CTX + GCSF	2.5	Y	6.69	Below level of detection	BU (2.4 × 4) CTX (50 × 4) ATG (1.25 × 4)	N	24	None	–	–
21	2023	19.0	CTX + GCSF	2.5	Y	6.70	ND	CTX (50 × 4) ATG (4.5^c^)	Y	19	None	–	Premature menopause
Median	2018	3.8		2.5		5.21				32			

ATG, antithymocyte globulin; BU, busulfan; C. diff, clostridium difficile infection; CTX, cyclophosphamide; FN, febrile neutropenia; GCSF, granulocyte colony‐stimulating factor; ND, not determined; PE, pulmonary embolism.

^a^ Peri‐transplant defined as starting from start of stem cell mobilization to 100 days after transplant.

^b^ Respiratory failure likely due to non‐infectious pneumonia.

^c^ ATG (thymoglobulin) given as 0.5 mg/kg on Day −3, 2.0 mg/kg on Day −2, and 2.0 mg/kg on day −1.

The shading was to signify an empty cell.

### Study outcomes

Disease severity prior to transplant was measured using the Myasthenia Gravis Foundation of America (MGFA) Clinical Classification.^24^ The primary efficacy endpoint was achieving MGFA Post‐intervention Status (PIS) of clinically stable remission (CSR, defined as without symptoms related to MG and without MG treatment for at least 1 year) or minimal manifestations‐0 (MM‐0; does not experience a limitation from MG and does not receive any MG treatment, but has weakness on examination), and then continuing to be in the CSR/MM‐0 until the most recent available follow‐up.^24^ MGFA scores were determined and documented at the time of assessment by the treating physician and extracted from patients' medical records. Patients were considered unevaluable for the primary efficacy endpoint if they were not followed for at least 1 year after the HSCT.

The primary safety endpoint was non‐relapse death, defined as death unrelated to MG, that is, death due to a non‐MG cause in a patient with no manifestations of MG or with minor manifestations of MG, that would not be expected to cause death. Secondary safety endpoint was transplant‐related death, defined as non‐relapse death occurring within 100 days post‐transplant. Basic descriptive statistics were used for both primary and secondary endpoints.

### Standard protocol approvals, registrations and patients consents

This study was reviewed and approved by the Research Ethics Board of the University of Calgary (REB21‐1963) and the Research Ethics Board of the Ottawa Hospital Research Institute (20220227‐01H). The respective REB from the University of Calgary and the University of Ottawa waived the requirement for informed consent.

## Results

### Demographics and MG activity before HSCT

Fifteen of the 21 patients (71%) were female. The median (range) age at diagnosis was 39 years (18–63 years) and the median (range) age at transplant was 48 years (25–65 years). The median (range) interval between diagnosis and transplant was 4.0 years (1.9–21.8 years), and the median number (range) of pretransplant non‐cholinesterase inhibitor treatments was 5 (3–7). Diagnosis of MG was based on combinations of clinical, electrophysiologic and/or serologic findings; 10 of 21 (48%) patients were AChR antibody‐positive and 6 of 17 (35%) were MuSK antibody‐positive (Table 3). Five patients were AChR‐negative, however, four of these patients underwent HSCT between 2001 and 2011 and did not have anti‐MuSK antibody testing (details on why these patients were thought to have MG are provided in Supplementary Material). Current standards of practice would advocate for testing for anti‐MuSK and LRP4 antibodies in those who are AChR antibody‐negative; however, this testing was not readily available in Canada at the time said patients were treated. All patients had severe disease (MGFA Class IVa, IVb, or V) when their MG was at its worst before the HSCT. Immediately before the transplant, severity ranged from mild refractory disease (Class IIA) to severe disease (Class IVa, IVb, or V) (Table 3). In all patients, MG was refractory to (or patients were intolerant of) multiple treatments (Table 3). Maximum daily doses of oral prednisone ranged from 25 mg to 70 mg. Nine patients had undergone thymectomy. Eight patients had a history of admission to an ICU for reasons related to MG, 7 of whom required intubation for respiratory support.

**Table 3:** Myasthenia gravis baseline disease status and treatment outcomes.

Patient number	MG therapies used prior to transplant																			
	Cholinesterase Inhibitors (Y/N)	Corticosteroids (Y/N)	Max daily dose of corticosteroids	Azathioprine (Y/N)	Mycophenolate (Y/N)	Rituximab (Y/N)	Eculizumab (Y/N)	IVIG (Y/N)	Plasmapheresis (Y/N)	Thymectomy (Y/N)	Antibody status	Worst MGFA between MG diagnosis and HCST	MGFA immediately prior to transplant	ICU admissions before HSCT	Best MGFA‐PIS	Time from HSCT to best MGFA‐PIS (years)	Primary efficacy endpoint met?	Follow‐up post‐transplant (years)	Death (Y/N)	Cause of death
1	Y	Y	70	N	N	Y	N	Y	Y	N	MuSK	V	IIb	3	CSR	1.4	Y	5.3	N	–
2	Y	Y	50	Y	Y	Y	N	Y	Y	N	AChR	IVb	IVb	0	Not Evaluable	2.9	N/A	3.8	N	–
3	Y	Y	60	Y	N	Y	N	Y	Y	N	MuSK	IVb	IIIb	0	CSR	1.1	Y	3.7	N	–
4	Y	Y	50	Y	Y	Y	N	Y	Y	N	MuSK	IVb	IIIb	0	CSR	1.3	Y	2.2	N	–
5	Y	Y	50	Y	N	Y	N	Y	Y	N	MuSK	IVb	IVb	0	Not Evaluable		N/A	0.1	Y	Respiratory failure^a^
6	Y	‐	‐	N	Y	N	N	Y	Y	Y	Other^c^	V	V	8	CSR	6.1	Y	21.9	N	–
7	Y	Y	25	N	Y	N	N	Y	Y	N	Other^c^	Unk	IIa	–	CSR	7.3	Y	9.4	Y	Colon Cancer
8	Y	Y	40	Y	N	N	N	Y	Y	Y	Other^c^	IVa	IVa	0	CSR	2.7	Y	12.2	N	–
9	Y	Y	60	Y	Y	N	N	Y	N	Y	AChR	IVb	IVb	0	CSR	6.2	Y	11.9	N	–
10	Y	Y	40	N	Y	N	N	Y	N	N	AChR	IVa	III	0	CSR	1.1	Y	11.9	N	–
11	Y	Y	60	Y	Y	N	N	N	Y	Y	Other^c^	IVb	IIIb	0	CSR	1.7	Y	8.6	Y	Sepsis
12	Y	Y	40	Y	Y	N	N	Y	Y	Y	AChR	IVa	IIIb	2	CSR	1.9	Y	8.2	N	–
13	Y	Y	60	Y	N	N	N	Y	Y	N	AChR	V	IIIa	2	CSR	1.1	Y	6.7	N	–
14	Y	Y	70	Y	Y	Y	N	Y	Y	N	AChR	IVa	IVa	0	Not Evaluable		N/A	0.1	Y	Sepsis
15	Y	Y	25	N	N	N	N	Y	Y	Y	AChR	IV	IVb	0	CSR	1.7	Y	3.7	N	–
16	Y	Y	30	N	Y	N	N	N	Y	N	AChR	V	IV	1	CSR	2.3	Y	4.1	N	–
17	Y	Y	30	N	Y	N	N	Y	Y	Y	Other^b^	IV	IVa	0	CSR	3.0	Y	3.0	N	–
18	Y	Y	50	N	N	Y	N	Y	Y	N	MuSK	IVa	IVa	0	CSR	1.1	Y	1.7	N	–
19	Y	Y	40	Y	N	Y	N	Y	Y	N	MuSK	IVb	IVa	0	MM‐1	1.1	N	1.6	N	–
20	Y	Y	Unk	Y	Y	Y	N	Y	Y	Y	AChR	V	IVb	1	MM‐3	1.0	N	1.0	N	–
21	Y	Y	Unk	N	Y	Y	Y	N	Y	Y	AChR	V	IVb	2	MM‐0	1.1	Y	1.1	N	–
Total											10 AChR 6 MuSK 5 Other^b^			19	15 CSR 1 MM‐0 1 MM‐1 1 MM‐3 3 NE		16 of 18 evaluable		4	
Median			50									IVb	IVa	0		1.7		3.8		

AChR, anti‐acetylcholine receptor antibody‐positive; HSCT, autologous hematopoietic stem cell transplant; IVIg, intravenous immunoglobulins; MGFA, Myasthenia Gravis Foundation of America score; MGFA‐PIS, Myasthenia Gravis Foundation of America Post‐intervention Status; MuSK, anti‐muscle‐specific kinase antibody‐positive; NE, not evaluable.

^a^ Respiratory failure likely due to non‐infectious pneumonia.

^b^ Seronegative for AChR and MuSK.

^c^ Seronegative for AChR, MuSK was not tested.

The darker shading that bottom of the table was meant to indicate an empty cell.

Patients with anti‐MuSK antibodies had an older median age of diagnosis (53 years) and transplant (55.5 years) compared to patients with anti‐AChR (38 and 42.5 years for diagnosis and transplant, respectively). Patients with anti‐AChR had 1:1 male to female ratio, while all patients included with anti‐MuSK were female.

### Stem cell mobilization, collection, and transplantation

Conditioning regimens are detailed in Table 2. Median (range) peri‐transplant hospitalization duration was 32.5 days (16–43 days). Grafts contained median (range) 4.8 × 10^6^ (2.3–9.2 × 10^6^) CD34+ cells/kg recipient weight. The CD3+ cell content was 1 × 10^4^ cells/kg or less for all patients (Table 2). During the transplant admission, 11 patients experienced febrile neutropenia, 3 patients developed CMV viremia that required treatment, and 2 experienced BK virus cystitis that required treatment. Three patients were admitted to ICU for a median (range) of 6 days (2–7 days) during their transplant admission; two had septic shock and the third had a pulmonary embolism.

### MG activity after HSCT

Patients had a median follow‐up of 3.8 years (range, 0.1–21.9 years) post‐transplant. Two patients were non‐evaluable for the primary efficacy endpoint due to early death (Pts. No. 5 and 14) and one was non‐evaluable due to concurrent myopathy (biopsy‐proven) (Pt. No. 2). Among the 18 evaluable patients, the primary efficacy endpoint (CSR or MM‐0 for ≥1 year and until end of follow‐up) was reached in 16 patients (88.9%) (Table 3) at a median (range) of 1.7 years (1.0–7.3 years). Median follow‐up for those achieving the primary efficacy endpoint was 6.7 years (range 1.0–21.9 years). Of the remaining evaluable patients, 1 achieved MM‐1 (no functional limitation associated with MG but there is some mild weakness on exam and the patient has received some form of immunotherapy within the past 1 year) and one achieved MM‐3 (no functional limitation associated with MG but there is some mild weakness on exam and the patient has received symptomatic therapy with a cholinesterase inhibitor and some form of immunotherapy within the last 1 year).^24^ Although these patients did not achieve the primary outcome, they still experienced an improvement of their MG (on fewer medications with improved MGFA clinical classification scores than before HSCT). The median time (range) to discontinuation of MG disease modifying therapies after HSCT for those patients in whom medications were stopped was 0.6 years (0.0–5.6 years). Four out of five eligible patients with anti‐MuSK antibodies achieved the primary outcome, compared to seven out of nine eligible patients with anti‐AChR.

Regarding the 3 non‐evaluable patients, in the 2 patients who died within 100 days post‐transplant, it is challenging to determine the efficacy of HSCT; however, the following were noted: Pt. No. 5 was pretransplant dependent on plasma exchange, cyclophosphamide, prednisone (20 mg/d), and pyridostigmine, and her dominant symptoms immediately pretransplant were orthopnea, dysarthria, and dysphagia. On Day 17 post‐transplant, she continued on a stable dose of pyridostigmine but required a lower prednisone dose (10 mg/d). She was 11 days from the last plasma exchange and had resolution of orthopnea, dysarthria, and dysphagia. The patient subsequently developed pneumonia requiring intubation and respiratory support, which was ultimately fatal and precluded assessment of MG status. Pt. No. 14 developed sepsis on day 10 after HSCT, which was too early to indicate whether there was an appreciable effect on his MG. Pt. No. 2 had persistent weakness after HSCT due to biopsy‐proven myopathy due to chronic steroid use. EMG after HSCT was not consistent with MG and acetylcholine receptor antibody, detectable pretransplant, was undetectable.

MG remissions after HSCT were sustained. Only one patient who had weaned immune‐suppressive medications after transplant required re‐initiation of pharmacotherapy for their MG (Pt. No. 20). She had near complete symptom relief and was tapered off azathioprine. However, 5 months post‐transplant, she developed recurrent muscle weakness and she was eventually diagnosed with Graves' thyroiditis. As her thyroid function improved, her weakness also improved. As of last follow‐up at 1.0 year post‐transplant, on steroid, intravenous immunoglobulin (IVIG), and pyridostigmine, her MGFA PIS was MM‐3. One patient who achieved CSR had disease worsening to MM‐0 between 4 and 5 years post‐transplant (Pt. No. 1), when she developed mild neck flexor weakness but did not require immunosuppressive therapy.

### Safety

The primary safety endpoint (death unrelated to MG) was reached in 4 of the 21 patients (19.0%) over the entirety of the follow‐up period. Two of these deaths occurred within 100 days after HCT. Thus, the transplant‐related mortality at 100 days was 9.5%. The two transplant‐related deaths were due to respiratory failure (Pt. No. 5) and Enterobacter sepsis (Pt. No.14) (details in Supplementary Material). Two late deaths occurred, with uncertain relation to the HSCT: Pt. No. 11 died of a saddle pulmonary embolus and sepsis at 8.7 years, and Pt. No. 7 died from colon cancer at 9.4 years after transplant. Several non‐fatal late transplant complications occurred in our cohort, including immune thrombocytopenia purpura (*n* = 2), hypothyroidism (*n* = 2), and premature menopause (*n* = 3).

**Figure 1:**
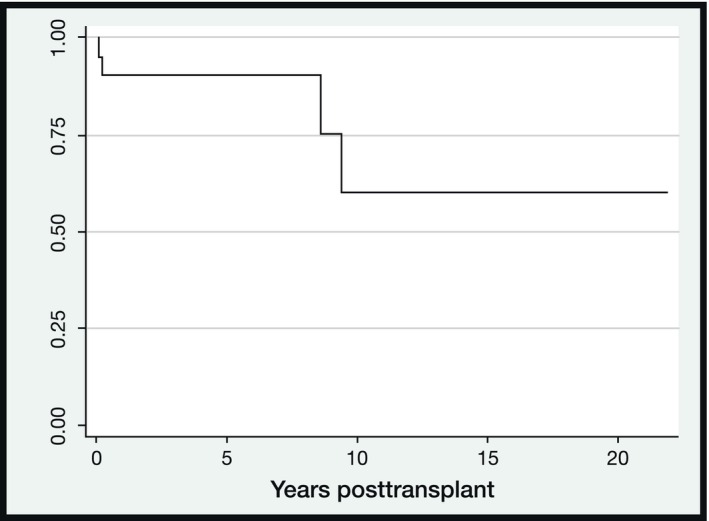
Kaplan–Meier curves showing overall survival.

Overall survival (OS) curve after HSCT is shown in Figure 1. Non‐relapse mortality curve is a mirror image of the OS curve, as all post‐transplant deaths were due to causes other than MG relapse.

## Discussion

We describe 21 patients with severe, rMG who were treated with HSCT over two decades. Patients underwent an immune‐depleting regimen of high‐dose chemotherapy and antithymocyte globulin with CD34 cell autografting. Among 18 evaluable patients, HSCT resulted in sustained MGFA‐PIS of CSR in 14 patients, MM‐0 in 1 patient and 1 patient who achieved CSR and subsequently had mild worsening after 4 to 5 years, classified as MM‐0.

The pretransplant chronic use of steroids complicated the MGFA‐PIS response evaluation for patient No.2 and No.19. Patient No.2 had biopsy‐proven myopathy from chronic exposure to steroids, as such she was considered non‐evaluable. Pt. No. 19 did not achieve the primary outcome as she remains on prednisone 5 mg/d, mainly for adrenal dysfunction. The patient's MG is in complete remission but is classified as MM‐1 due to prednisone use.

The transplant‐related mortality in our series was 9.5%, which is not unexpected, given how medically complex patients become after years of rMG and multiple rounds of anti‐MG therapy. It is important to compare this to mortality for rMG; however, it is challenging to precisely derive a figure for mortality among patients with refractory MG because (1) there is no consensus definition of refractory MG, (2) there is heterogeneity in the studies of rMG given that there is no standard definition, and (3) the number of available treatments is growing rapidly. Within these limitations, one may estimate the mortality of rMG as double that of non‐refractory MG, based on a study of Korean patients with refractory MG that showed an adjusted HR of 2.49 (95% CI 1.26–4.94).^7^ The mortality rates of MG have declined over the last several decades and estimates of mortality from any degree of MG range from 2.1–9%, which includes both rMG and non‐rMG.^25^, ^26^ Within these limitations, it may be reasonable to suggest that the rates of mortality from HSCT are not that dissimilar from those of rMG but more robust studies on the mortality associated with rMG are needed. In the treatment of multiple sclerosis (MS) with HSCT there has been a trend toward decreasing mortality, with several recent cohorts demonstrating <1% mortality^27^, ^28^, ^29^, ^30^, ^31^, ^32^, ^33^ One possible explanation is that in the treatment of MS, there has been a shift in patient selection from those with advanced disability to those earlier on in their disease course with less exposure to chronic treatments.^28^, ^29^, ^30^, ^31^ Factors associated with increased treatment‐related mortality with the use of HSCT for the treatment of MS include older age, baseline degree of disability, and high‐intensity transplant conditioning regiments.^34^, ^35^ The majority of the patients in our cohort were exposed to high‐intensity conditioning regimen as a part of their HSCT. A low intensity regimen is where cyclophosphamide is used with or without anti‐thymocyte globulin. In contrast, a high‐intensity condition regimen is one that includes busulfan or total‐body irradiation.^35^ Therefore, the three factors which may be modified to improve TRM in our cohort would be selecting younger patients and patients with lower degree of disability at the time of transplant, or using lower intensity regimens. Patients with MG can also experience disability through the cumulative effect of corticosteroids which includes hypertension, obesity, hyperglycemia, myopathy, and osteoporosis leading to increased risk of fragility fractures.^36^ As is described in the vignette for patient no. 5, these effects can have important and detrimental effects on mobility, functional status, and mortality. Future high quality, prospective studies on the use of HSCT in the treatment of MG may want to select patients with rMG earlier on in their disease course to mitigate both the effect of increasing age on TRM but also to avoid the effects of chronic corticosteroid use. Undergoing HSCT before accruing disability from MG therapies may confer lower risk of transplant complications.

Two patients (No. 7 and 11) died several years after their transplant from sepsis and from colorectal cancer, respectively. There is uncertainty regarding the association between HSCT and these deaths. Nevertheless, patients who receive HSCT for a malignancy have reduced overall survival compared to the general population due at least in part to new malignancies.^37^

Although HSCT timing may contribute to safety outcomes, it did not contribute to efficacy outcomes in our cohort. The primary outcome was achieved for patients with a range of time intervals between diagnosis and HSCT (1.9–21.8 years), as well as a variety of pre‐transplant therapies (Table 3). In contrast to cancer and MS, where a delay in HSCT results in poorer outcomes, MG appears to be responsive to HSCT even in patients with advanced MG who have failed multiple other immune therapies.

Healthcare costs are an important factor when considering the practicality of HSCT in the landscape of novel MG therapies. The yearly cost of Eculizumab is at estimated at $855,400 (USD) while the estimated yearly cost of Efgartigimod is $692,700 (USD) and patients remain on therapy indefinitely.^38^ HSCT is associated with a mean, one‐time cost of $108,577 (USD) per patient (range $56,327–277,119); estimated between 2017 and 2019 in the United States.^39^ Patients treated with eculizumab and efgartigimod are also generally treated concurrently with other disease modifying therapies in comparison to HSCT in which the majority of patients achieved CSR, and those who remained on therapy in our cohort were on modest doses of prednisone. Novel MG therapies are available as infusions or subcutaneous injections which are performed by healthcare providers often in infusions centers or clinics, requiring visits weekly, biweekly, or monthly. This is in contrast to HSCT, which requires a hospitalization and multiple peri‐transplant visits; however, only annual follow‐up thereafter, thus over years decreasing the total number of visits required to healthcare facilities.

Our study has limitations. One is the retrospective nature of the study, associated with a lack of prospective evaluation autoantibody levels after HSCT. Another major limitation is that patients included in the study were not treated with novel MG therapies that are now often used for severe or rMG, except for one patient who failed eculizumab (No. 21). Eculizumab is a humanized monoclonal antibody that targets the terminal complement protein, C5.^40^ The MGFA‐PIS reported in the interim analysis of the REGAIN trial suggested that 56.0% achieved MM or PR and 74.1% had improvement in their disease status.^41^ MGFA‐PIS was not reported in the extension trial.^42^ Although it would not be appropriate to make direct comparisons between our small cohort and this trial, it is apparent that none of the patients in the REGAIN trial achieved disease remission or were able to remain in treatment‐free remission, which was the case for the majority of our patients. Whereas with HSCT we expect near normal immune reconstitution after auto‐HCT, ongoing therapy with eculizumab would lead to a persistent immune defect against encapsulated bacteria, among other pathogens. Efgartigimod is a human IgG antibody that functions to reduce the recycling of auto‐inflammatory IgG molecules by inhibiting the binding of IgG to the neonatal Fc receptor.^5^ In the ADAPT trial, not all patients were refractory to treatment; 23 out of 167 (13.8%) had not received either steroid or non‐steroid immunosuppression, 40 out of 167 (24.0%) did not receive steroid, and 65 out of 167 (38.9%) did not receive any non‐steroid immunosuppression.^5^ Although efgartigimod is effective at improving MG‐ADL and QMG scores in patients with severe MG, it is unknown whether similar efficacy would be seen in patients with refractory disease, like those included in our cohort. Although no studies of quality of life (QoL) are available, QoL after autologous HCT for malignancy appears to return to population norm by 1‐year post‐transplant.^43^

Our cohort was heterogenous in the treatments that they received prior to transplant. This may have implications on their disease status prior to HSCT, particularly as many received their transplant prior to the availability of rituximab in our region (although 9 out of 20 patients failed rituximab) and none received efgartigimod. However, the heterogeneity reflects the real‐world experience in treating MG and the improvement in disease control resulting in sustained CSR/MM‐0 in of the cohort despite the heterogeneity speaks to the efficacy of HSCT in rMG. The HSCT conditioning regimens were not completely uniform among our cohort (Table 2) and the low numbers of alternative conditioning regimens makes it difficult to compare one to another. Other limitations were the incomplete information for some patients and, although this is the largest cohort of MG patient treated with HSCT, it is still a small sample size which may have implications on the generalizability of the data. The diagnosis of MG was made based on a combination of clinical, serological, and/or electrophysiological findings in our patients who came from multiple provinces, multiple healthcare systems, over multiple decades before electronic medical records were available. We did not have results for electrophysiologic testing around the time of diagnosis for one of the patients who was seronegative (No 17); however, their clinical presentation was consistent with MG (see Supplemental Material).

## Conclusion

Sixteen out of 18 evaluable patients with rMG who underwent HSCT met the primary efficacy outcome of MGFA‐PIS CSR or MM‐0, which was sustained until most recent follow‐up. Two patients died in the peri‐transplant period related to toxicity from HSCT. The toxicity might be better tolerated if patients were treated earlier their disease course, prior to accumulating disability and comorbidities or if the transplant regimen chosen was less toxic. Another advantage of HSCT is the reduced healthcare costs compared to novel anti‐MG treatments. High‐quality, Phase 2 prospective studies are warranted for the evaluation of HSCT as a treatment for rMG.

## Funding Information

There is no financial or material support to report for this work.

## Conflict of Interest

The authors declare no conflicts of interest.

## Author Contributions

Benjamin Beland was responsible for data extraction, data analysis, and the writing of the first manuscript draft. Jan Storek was responsible for study design, data analysis, and manuscript revisions. Liam Quartermain was responsible for data extraction and manuscript revisions. Christopher Hahn, C. Elizabeth Pringle, Pierre Bourque, Michael Kennah, Natasha Kekre, Christopher Bredeson, David Allan, Kareem Jamani, and Christopher White were responsible for data contribution and manuscript revisions. Harold Atkins was responsible for study design, data extraction, and manuscript revisions.

## Supporting information


Table S1.

Appendix S1.

## Data Availability

The authors agree to supply anonymized data regarding the study cohort upon reasonable requests.
