# Supplementary material for: Refractory myasthenia gravis treated with autologous hematopoietic stem cell transplantation
Source: Ann Clin Transl Neurol. 2024 Dec 31;12(1):56–68. doi: 10.1002/acn3.52246 (PMC11752101; doi:10.1002/acn3.52246)
Supplement: Supplementary file 2 — Appendix S1. [file ACN3-12-56-s002.docx]

**Supplementary Material**

**Seronegative patients – Vignettes showing why the patients were diagnosed with MG**:

Patient 6 – EMG inconclusive

Patient 7 – EMG supported diagnosis

Patient 8 – EMG supported diagnosis

Patient 11 – EMG supported diagnosis

Patient 17 – no EMG on record

**Patients who died within 100 days after HSCT – Vignettes showing that pretransplant the patients were very debilitated**

Patient 5 – Debilitated by preexisting lung damage, multiple MG crises, and severe steroid toxicity

Patient 14 – Debilitated by multiple MG crises

**Patient 6**

Patient 6 was diagnosed with myasthenia 57 month prior to HSCT based on clinical symptoms and findings. She had a single-fibre EMG performed at a separate institution that was consistent with myasthenia gravis. She underwent thymectomy 12 months after diagnosis resulting in a 3-month remission of myasthenia.

She was admitted to ICU 17 months after diagnosis for a myasthenic crisis while on treatment with pyridostigmine and IVIG. The myasthenia improved with plasmapheresis, 30 mg prednisone daily, and pyridostigmine. Additional hospitalizations for myasthenic crisis occurred 3 month, 23 months, and 29 months later. Maintenance therapy with chronic plasmapheresis and varying doses of prednisone were used to control myasthenia. Ongoing symptoms included bilateral fatigable ptosis, facial weakness, neck weakness and mild weakness of the proximal upper and lower limb muscles. During this period, there was intolerance to a trial of azathioprine, an anaphylactoid reaction to IVIG and an admission for methicillin sensitive staphylococcus aureus and klebsiella bacteremia.

Forty-eight months after diagnosis, operative exploration for a remnant thymus was performed but no additional tissue was discovered. Five additional hospitalizations, including ICU admissions for myasthenic crises, sepsis, pneumonia and C. Difficile colitis and the need for mechanical ventilation, occurred between 50 and 54 months after diagnosis despite ongoing treatment with plasmapheresis, steroids, mycophenolate mofetil and pyridostigmine.

Hematopoietic stem cells were mobilized with cyclophosphamide and filgrastim and a graft was successfully collected 54 months after diagnosis. She was subsequently readmitted and required mechanical ventilation for myasthenia respiratory failure three times before undergoing CD34 selected autologous hematopoietic stem cell transplantation 57 months after diagnosis.

Mechanical ventilation and ICU care was required in twice in the second post-HSCT month, once in third post-HSCT month for respiratory muscle weakness, and once in the fourth post-HSCT month precipitated by a diarrheal illness. Plasmapheresis was used episodically during some of these admissions. She was admitted and placed on BiPAP 22 months after HSCT for breathing issues felt to be due to vocal cord scarring and recovered following a brief course of prednisone and pyridostigmine. She has been free of myasthenia symptoms and has not required hospitalization for myasthenia since. She had a normal EMG 51 months after HSCT.

**Patient 7**

Patient 7 presented with diplopia and other ocular and bulbar symptoms leading to a clinical diagnosis of myasthenia, 9 years prior to HSCT. Anti-acetylcholine receptor autoantibodies were never detected in 8 samples drawn between diagnosis and hematopoietic stem cell transplantation. Testing for anti-muscle specific kinase autoantibodies was not available. Treatment with pyridostigmine was brief because of intolerable headaches. Prednisone was started.

EMG performed in 2 years after diagnosis reported “*no definitive evidence of a defect in neuromuscular transmission*”. Repeat EMG 3 months later were limited in scope and were felt to be normal within the limitations of the study. Evoked potentials performed 3 years after the clinical diagnosis were abnormal reporting: “*The interpeak latency between the first and last components on the left side is just beyond the normal range suggesting the possibility of ipsilateral brainstem dysfunction.”*  EMG performed in 4 years after the clinical diagnosis reported: “*These electrophysiologic tests are abnormal. They are consistent with the clinical diagnosis of myasthenia gravis*.”

EMG performed 10 years after diagnosis, two months prior to hematopoietic stem cell mobilization and collection, reported: ”…*definite evidence for defective neuromuscular transmission consistent with the patient's clinical diagnosis of generalized myasthenia gravis. There was no electrical evidence of an underlying neuropathy.*”

**Patient 8**

13 years prior to transplant, patient 8 developed symptoms of fatigable weakness and clinical finding consistent with myasthenia gravis. Serum antibody testing did not AChR antibodies and anti-MuSK antibodies were not performed at the time.

Nerve conduction and EMG at the time demonstrated decrement of 19.3% using 3Hz repetitive stimulation and single-fibre EMG demonstrated jitter, consistent with dysfunction of neuromuscular junction transmission and myasthenia gravis.

**Patient 11**

Patient 11 presented at a distant hospital with subacute ptosis, horizontal diplopia, dysphagia, chewing fatiguability, slurred speech with talking, neck weakness 88 months prior to HSCT. An ophthalmologist made the diagnosis of myasthenia gravis and referred her to a neurologist. Anti-acetylcholine receptor antibody testing was performed by the referring physician at that time but the results were not available at the treating center.

She was seen in consultation in 83 months prior to HSCT at which time she had been on pyridostigmine and azathioprine for 3 months. The myasthenic symptoms responded to treatment with pyridostigmine. The neurologic examination at the time of consultation was consistent with generalized myasthenia gravis including bilateral fluctuating ptosis, fatigable diplopia, and fatigable weakness of the upper limbs.

Single fiber EMG performed in 82 months prior to HSCT were strongly in keeping with myasthenia.

*Single fiber EMG: The left frontalis muscle was steadied. Nine fiber pairs were available for analysis. Five of these showed very abnormal gitter, with values ranging from 112 to 158 microseconds. The mean value for the group was 87.8 microseconds, also quite abnormal. INTERPRETATION: Normal right facial repetitive stimulation, borderline abnormal right accessory repetitive stimulation study, markedly abnormal single fiber EMG of the left frontalis. The single fiber EMG is certainly strongly in keeping with a neuromuscular junction disorder such as myasthenia gravis.*

Repeat Anti-acetylcholine receptor antibody testing was negative 1 month prior to hematopoietic stem cell transplantation although she was receiving plasmapheresis during that time period.

**Patient 17**

Patient 17’s symptoms began slowly with fatigability and vision changes more than 20 years prior to HSCT. When myasthenia is active, her symptoms include trouble chewing, difficulty holding her head up, and poor endurance. Her symptoms improved with pydridostigmine. She underwent a thymectomy 19 years prior to HSCT and was not bothered by myasthenia for about 6 years. She started immunosuppressive therapy in 12 years prior to HSCT as myasthenia became more symptomatic. She was hospitalized for myasthenic crisis twice over the 20-year course but never required intubation. She intermittently required treatment with prednisone for flares of myasthenia. She was treated with pyridostigmine, mycophenolate mofetil, and weekly IVIG for until a year prior to HSCT when the myasthenia became more active and she was hospitalized for a myasthenic crisis. She was started on high dose prednisone and switched from IVIG onto plasmapheresis.

She developed a mixed connective tissue disease with features of systemic lupus erythematosus and scleroderma 5 years prior to HSCT, characterized by cutaneous photosensitive lupus-like rash on her face and upper torso, arthralgias, sicca syndrome and non-scarring alopecia, gastrointestinal reflux, raynaud’s phenomenon, dilated nailbed capillaries, teleangiectasia and calcium deposits. She was positive for anti-RNP and anti RO-60 autoantibodies and an ANA with a speckled pattern and a titre of 1:640.

She was referred by her rheumatologist because of myasthenia and mixed connective tissue disease. She was first seen in consultation for hematopoietic stem cell transplantation 15 months prior to HSCT at age 53. Anti-acetylcholine receptor autoantibodies testing at the transplant center was repeatedly negative for anti-acetylcholine receptor and anti-musk autoantibodies although she was receiving frequent plasmapheresis. Examination by a neurologist at our center in 3 months prior to HSCT demonstrated ptosis with brief upward gaze, fatigability of neck extensors after about 10 seconds of upward gaze and mild weakness of shoulder abductors and hip flexors. The symptom history and examination supported the diagnosis of myasthenia.

Note: The information available on this patient is limited due to MG diagnoses more than 2 decades pre-HSCT, outside of a hospital, in a different province than where the HSCT was performed, before the era of electronic medical records, and HSCT during Covid-19 pandemic (EMG testing was restricted by pandemic measures).

**Patient 5** (Debilitated by preexisting lung damage, multiple MG crises, and steroid toxicity)

Pt. No. 5 was a 56-year-old woman diagnosed with MG in 2020, who had positive anti-MuSK antibodies. She was hospitalized for organizing pneumonia in 2021 and recovered only partially. She was on pyridostigmine and prednisone (up to 50mg daily) for 2 years prior to transplant. She underwent HSCT after 6 hospitalizations for MG crisis, 2 hospitalizations for Covid19 pneumonia, 2 years of treatment with prednisone 20-50 mg/d and having failed rituximab and not tolerated IVIG. Immediately pretransplant, she required cyclophosphamide 1.2 g iv every 2-3 weeks in addition to prednisone, plasma exchanges, and pyridostigmine.

She had complications from chronic corticosteroid use including blindness from advanced steroid-induced cataracts, steroid-induced osteoporosis with pretransplant compression fractures of 4 thoracic vertebrae and she developed compression fractures of 2 lumbar vertebrae peri-transplant, rendering her virtually bedridden, despite MG symptoms improved while tapering anti-MG treatments. Her immobility contributed to the development of pneumonia (initially infectious and later non-infectious organizing pneumonia on top of the preexisting residual organizing pneumonia), resulting in admission to the ICU and ventilator support for respiratory failure from which she ultimately died.

**Patient 14** (Debilitated by multiple MG crises)

Patient 14 developed difficulties swallowing, talking, and walking during the 28 months prior to HSCT at age 63. An EMG performed was consistent with the diagnosis of generalized myasthenia. Serum anti-acetylcholine receptor autoantibodies were positive. Chest CT did not demonstrate a thymoma. Over the 2 years following diagnosis, he had 5 admissions to the ICU for myasthenic crises although did not require intubation. He received treatment with pyridostigmine, chronic plasmapheresis, prednisone, mycophenolate, azathioprine, rituximab, and monthly intravenous cyclophosphamide with minimal impact on symptoms or impairments caused by myasthenia. His comorbidities included medication-controlled hypertension and benign prostatic hypertrophy. Pre-transplant baseline echocardiography showed concentric left ventricular remodeling with an ejection fraction of 73%. Persantine myocardial perfusion scanning and Holter monitoring was unremarkable. He was unable to reproducibly perform pulmonary function testing because of myasthenia.

Hematopoietic stem cells were mobilized with cyclophosphamide and filgrastim without complication. A hematopoietic stem cell graft containing 2.3x10^6^ CD34 cells/kg recipient weight was collected by leukapheresis and cryopreserved. He received BEAM conditioning followed by infusion of the stem cell graft. He developed febrile neutropenia due to Enterobacter cloacae and pseudomonas aeruginosa bacteremia on day 3 and was started on broad-spectrum antibiotics. Computerized tomogram of the chest demonstrated dense bilateral consolidation throughout both lungs on day 4. Hemoptysis and sepsis developed on day 5 resulting in intubation, mechanical ventilation, vasopressors, and other supportive measures being instituted following transfer to intensive care. Sustained low-efficiency dialysis was started for anuric renal failure. Comfort measures were instituted following a goals of care discussion because of ongoing clinical deterioration from acute respiratory distress syndrome and sepsis on day 10. The patient succumbed shortly thereafter.
